# Lipoprotein glomerulopathy with markedly increased arterial stiffness successfully treated with a combination of fenofibrate and losartan: a case report

**DOI:** 10.1186/s12882-024-03612-z

**Published:** 2024-05-20

**Authors:** Junichiro Kato, Hideo Okonogi, Go Kanzaki, Haruki Katsumata, Yasuyuki Nakada, Makoto Sagasaki, Kazumasa Komine, Kenji Ito, Takao Saito, Akira Matsunaga, Koh Tokutou, Kazuho Honda, Nobuo Tsuboi, Takashi Yokoo

**Affiliations:** 1 Division of Nephrology and Hypertension, Department of Internal Medicine, Atsugi City Hospital, 1-16-36, Mizuhiki, Atsugi City, Kanagawa, 243-8588 Japan; 2 Division of Nephrology and Hypertension, Department of Internal Medicine, The Jikei University School of Medicine, Tokyo, Japan; 3 Department of Pathology, Atsugi City Hospital, Kanagawa, Japan; 4 Division of Nephrology and Rheumatology, Department of Internal Medicine, Faculty of Medicine, Fukuoka University, Fukuoka, Japan; 5 Sanko Clinic, Fukuoka, Japan; 6 General Medical Research Center, Faculty of Medicine, Fukuoka University, Fukuoka, Japan; 7 Department of Anatomy, Showa University School of Medicine, Tokyo, Japan

**Keywords:** Lipoprotein glomerulopathy, Apolipoprotein E, Oil Red O, Sudan IV, Triglyceride, Fibrate, Arterial stiffness, PWV, CAVI

## Abstract

**Background:**

Lipoprotein glomerulopathy (LPG) is a apolipoprotein E (ApoE)-related glomerular disease and has been associated with type III hyperlipidemia. Without appropriate treatment, chronic kidney disease (CKD) caused by LPG progresses, and approximately half of the patients develop end-stage kidney disease within 1–27 years of disease onset. However, few studies have highlighted the clinical course of cardiovascular diseases (CVDs) in patients with LPG. Herein, we report the first case of LPG in which the CVD risk was assessed using arterial stiffness.

**Case presentation:**

A 32-year-old Japanese man was referred to our hospital due to persistent proteinuria. Kidney biopsy showed markedly dilated capillary lumens containing pale-stained thrombi, which stained positively with Oil Red O. Electron microscopy revealed the presence of thrombi in the capillary lumen with low electron density and vacuoles of various sizes in part of the thrombi. Toluidine blue and Sudan IV stains were used to stain the thin sections of Epon-embedded tissue samples for electron microscopy. Sudan IV-positive droplets were observed in the capillary lumens, vascular walls, and cytoplasm of tubular cells. Increased serum ApoE concentration was observed. Liquid chromatography-tandem mass spectrometry of laser-microdissected glomeruli from paraffin sections revealed an increase in ApoE. Direct deoxyribonucleic acid sequencing of ApoE revealed a heterozygous ApoE Sendai mutation (Arg145Pro). The patient was finally diagnosed with LPG with heterozygosity for ApoE-Sendai mutation (Arg145Pro). Notably, at the time of diagnosis, he had markedly increased arterial stiffness for his age. Arterial stiffness was measured using brachial-ankle pulse wave velocity (baPWV), which was equivalent to that of a 56-year-old man. After three months of treatment with fenofibrate and losartan, a significant reduction in proteinuria was achieved along with an improvement in baPWV. Furthermore, these effects were maintained despite the lack of decrease in serum ApoE levels.

**Conclusion:**

Herein, we report the case of a patient with LPG with markedly increased arterial stiffness at the time of diagnosis, in whom combination therapy with fenofibrate and losartan successfully improved proteinuria and arterial stiffness. To the best of our knowledge, this is the first case report of LPG in which CVD risk was assessed using arterial stiffness.

## Background

Lipoprotein glomerulopathy (LPG) is a apolipoprotein E (ApoE)-related glomerular disease that was first reported as a glomerular disease associated with type III hyperlipidemia [1]. LPG is caused by a mutation in the ApoE gene and is histologically characterized by dilated glomerular capillaries with lipoprotein thrombi showing lamella formation instead of foam cells [2, 3]. Among the known causative genetic mutations for LPG, ApoE Sendai is the most common in Japan [4]. The elucidated pathogenic mechanism of LPG has been summarized here [5]. First, the property of the mutant ApoE protein leads to easy self-aggregation, loss of its ability to bind to low-density lipoprotein (LDL) receptor, and retention of its binding ability to heparan sulfate proteoglycan. Second, intrinsic glomerular features, such as conduction to thrombus formation through the tortuous capillary structure and mutual attraction between the negatively charged basement membrane and positively charged mutant protein, may lead to self-aggregation. Third, changes in the phagocytic function of macrophages due to Fc receptor gamma chain deficiency may lead to LPG [6]. In addition, hyperlipidemia may play a direct or indirect detrimental role in kidney pathology through inflammation, reactive oxygen species, endogenous electrical stress, and other pathways. Furthermore, lipid accumulation inside kidney cells contributes to the development of chronic kidney disease (CKD), irrespective of the presence of hyperlipidemia [7]. Without appropriate treatment, CKD caused by LPG progresses, and approximately half of the patients develop end-stage kidney disease within 1–27 years of disease onset [2].

Accumulating evidence suggests an association of dyslipidemia with the risk of CKD progression and cardiovascular outcomes in patients with CKD [8]. However, few studies have highlighted the clinical course of cardiovascular diseases (CVDs) associated with LPG [5].

Conventional risk factors for CVD, such as age, hypertension, diabetes mellitus, dyslipidemia, smoking, uric acid, obesity, and CKD, increase the arterial stiffness of medium to large arteries, which can be measured using brachial-ankle pulse wave velocity (baPWV) [9] or cardio-ankle vascular index (CAVI) [10]. Therefore, arterial stiffness is a useful marker for predicting future cardiovascular events in the absence of a history of CVD, independent of conventional risk assessment models [9, 10].

Herein, we report the first case of LPG with markedly increased arterial stiffness at the time of diagnosis in whom combination therapy with fenofibrate and losartan successfully improved both proteinuria and arterial stiffness.

## Case presentation

A 32-year-old Japanese man was referred to our hospital due to persistent proteinuria, which had been noted during health checkups since his twenties. His past medical history was otherwise unremarkable. The patient had no history of smoking. His younger brother, an identical twin, visited another hospital with the same complaint of proteinuria. His grandmother had kidney disease. At the first visit to our hospital, the patients’ urine protein-to-creatinine ratio (uPCR) was 2.2 g/gCreatinine (gCr), urine sediment red blood cell (RBC) was < 1 /high-power field (HPF), and estimated glomerular filtration rate (eGFR) was 97.8 mL/min/1.73 m^2^. Proteinuria was also observed in subsequent outpatient visits (maximum 2.7 g/gCr), and he was admitted to our hospital five months after the initial visit.

At the time of kidney biopsy, his blood pressure (BP) was 129/90 mmHg. His body weight was 61.5 kg and his body mass index (BMI) was 21.8 kg/m^2^. The patient neither had pitting edema of the lower extremities nor any xanthomas. Laboratory findings were as follows: urine protein excretion, 2.3 g/day; uPCR, 1.2 g/gCr; urine sediment RBC, < 1/HPF; urine N-acetyl-β-D-glucosaminidase, 2.1 U/gCr; urine β2 microglobulin, 101 μg/gCr; eGFR, 95.5 mL/min/1.73 m^2^; serum albumin, 4.0 g/dL; triglyceride (TG), 178 mg/dL; high-density lipoprotein cholesterol (HDL-C), 46 mg/dL; and LDL-C, 154 mg/dL. In addition, his serum ApoE was 7.6 mg/dL (reference range: 2.7–4.3 mg/dL). The lipid profiles, including those of apolipoproteins before and after 12 months of treatment, are summarized in Table 1. In the context of steno-stiffness examination for CVD risk assessment [9], the ankle-brachial index was 1.2, while the baPWV was 14.3 m/s (BP, 123/70 mmHg at measurement), which was equivalent to that of a 56-year-old man.

**Table 1:** Lipids profiles before and after 12 months of treatment

	Before	After	Reference range
TG (mg/dL)	178	56	40–234
HDL-C (mg/dL)	46	76	38–90
LDL-C (mg/dL)	154	103	65–163
Apo A-I (mg/dL)	123	145	119–155
Apo A-II (mg/dL)	30.8	57.1	5.9–35.7
Apo B (mg/dL)	114	70	73–109
Apo C-II (mg/dL)	6.5	6.1	1.8–4.6
Apo C-III (mg/dL)	10.7	8.3	5.8–10.0
Apo E (mg/dL)	7.6	12.8	2.7–4.3
RLP-C (mg/dL)	13.8	2.3	-7.5
Lp(a) (mg/dL)	8.5	n.d	-30

Abbreviations: *TG* triglyceride, *HDL-C* high-density lipoprotein cholesterol, *LDL-C* low-density lipoprotein cholesterol, *Apo* apolipoprotein, *RLP-C* remnant-like particle cholesterol, *Lp(a)* lipoprotein (a), *n.d.* no data

**Fig. 1:**
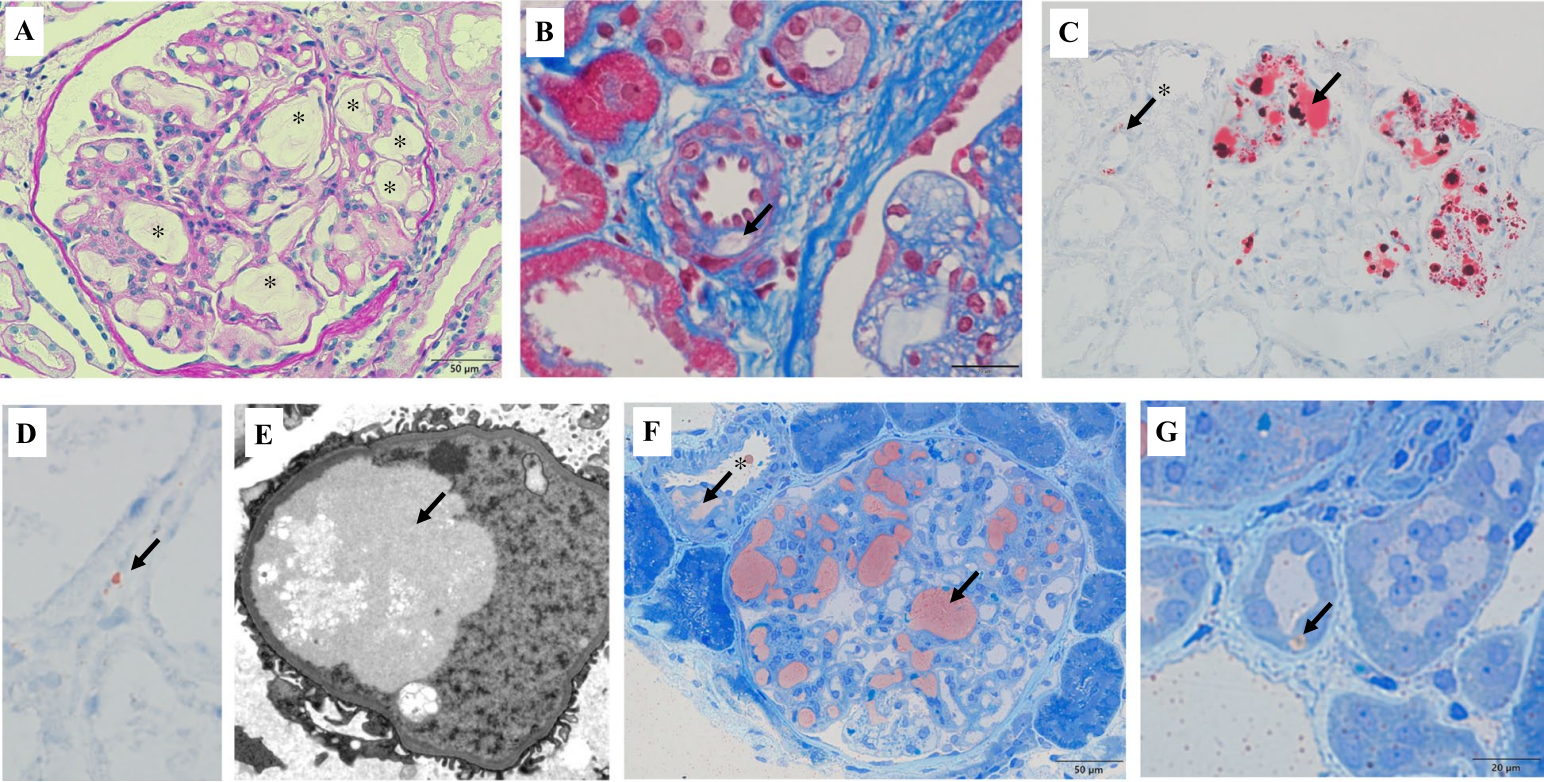
Microscopy findings of the kidney biopsy. **A** Capillary lumens are markedly dilated and occupied by pale-stained thrombi with a vaguely laminated appearance (asterisks) (PAS stain; original magnification × 400). **B** A pale-stained deposit is observed in the afferent arteriole wall (arrow) (Masson stain; original magnification × 1000). **C**,** D** Oil Red O staining showing several lipid droplets within thrombus-like substances in the glomeruli (C, arrow), tubular cytoplasm (C, arrow with asterisk) and peritubular capillaries (D, arrow) (Oil Red O staining; original magnification × 400). **E** Electron micrograph showing thrombi in the capillary lumen with low electron density and vacuoles of various sizes in parts of the thrombi. Partial effacement of the foot processes of epithelial cells is evident near the thrombi (original magnification × 5000). **F**,** G** Toluidine blue and Sudan IV staining of thin sections of Epon-embedded tissue samples for electron microscopy. Sudan IV-positive droplets are observed in the capillary lumen (F, arrow), vascular wall (F, arrow with asterisk), and cytoplasm of tubular cells (G, arrow) (F, original magnification × 400; G, original magnification × 1000). Abbreviation: PAS, Periodic acid-Schiff

Subsequently, a kidney biopsy was performed. The light microscopy specimen contained 71 glomeruli, two of which exhibited adhesion. However, no global glomerulosclerosis or glomeruli with crescent formations were observed. The percentage of interstitial fibrosis/tubular atrophy in the cortical area was minimal (< 5%). Majority of the glomeruli were enlarged and the capillary lumens were markedly dilated and occupied by pale-stained thrombi with a vaguely laminated appearance (Fig. 1A). A pale-stained deposit was observed in the afferent arteriolar wall (Fig. 1B). Oil Red O staining revealed several lipid droplets within the thrombus-like substances in the glomeruli, tubular cytoplasm, and peritubular capillaries (Fig. 1C and D). Foam cells were observed in the interstitial area, but not in the glomeruli.

Routine immunofluorescence analysis showed slight C3c deposition at the vascular pole of the glomeruli. However, no remarkable deposition of immunoglobulins or other complements were observed.

Electron microscopy revealed the presence of thrombi in the capillary lumen with low electron density and vacuoles of various sizes in parts of the thrombi. Moreover, partial effacement of the foot processes of epithelial cells was observed near the thrombi (Fig. 1E).

The intrarenal localization of lipoprotein deposition was further evaluated using toluidine blue and Sudan IV stained thin sections of Epon-embedded tissue samples for electron microscopy [11]. Sudan IV-positive droplets were observed not only in the capillary lumens, but also in the vascular wall and cytoplasm of tubular cells (Fig. 1F and G).

**Fig. 2:**
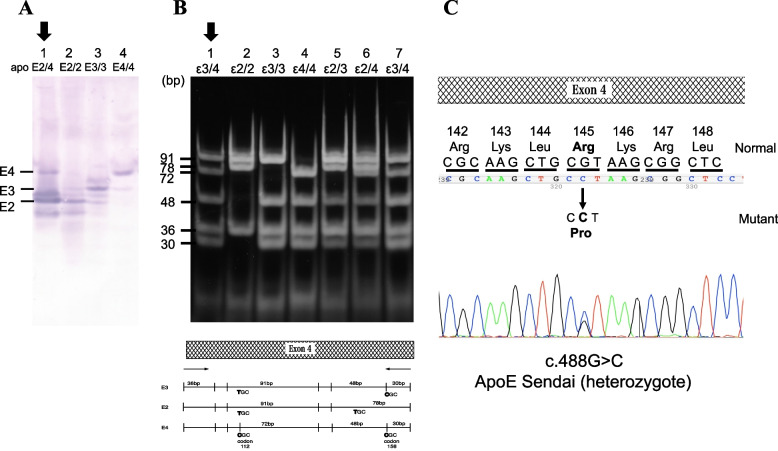
Phenotype and genotype analysis, and direct sequence of genomic DNA. **A** ApoE phenotype was determined using isoelectric focusing polyacrylamide gel electrophoresis. Lane 1: ApoE2/4 (patient); lane 2: ApoE2/2; lane 3: ApoE3/3 (wild type); lane4: ApoE4/4. **B** ApoE genotype determined using restriction fragment length polymorphism. The PCR-amplified ApoE DNA, including codon 145, was digested with HhaI and subjected to polyacrylamide gel electrophoresis. Lane 1: PCR sample (patient); lane 2: ε2/2; lane3: ε3/3 (wild type); lane4: ε4/4; lane5: ε2/3; lane6: ε2/4; lane7: ε3/4. The lower panel shows a schematic illustration of ApoE2, ApoE3, and ApoE4 genes with restriction site of HhaI. **C** Sequence of genomic DNA from the patient with LPG. The normal ApoE allele contains the sequence CGT (arginine) at codon 145, whereas the mutant ApoE Sendai contains CCT (proline) at the same codon. Abbreviations: ApoE, apolipoprotein E; PCR, polymerase chain reaction; DNA, deoxyribonucleic acid

The ApoE phenotype, analyzed using isoelectric focusing polyacrylamide gel electrophoresis, as previously described [12], was ApoE2/4 (Fig. 2A). The ApoE genotype, determined by restriction enzyme Hha I, was ε3/4 (Fig. 2B). Thus, a discrepancy was observed between the ApoE phenotype and genotype. Direct deoxyribonucleic acid (DNA) sequencing of ApoE revealed a heterozygous ApoE-Sendai mutation (Arg145Pro) (Fig. 2C).

Next, laser-microdissected glomeruli from paraffin sections were analyzed using liquid chromatography-tandem mass spectrometry (LC–MS/MS), as previously described [13]. LC–MS/MS revealed a significant increase in ApoE in the glomeruli (Table 2).

**Table 2:** Liquid chromatography-tandem mass spectrometry

	Normal control	this case	Fold (Case/Control)
ApoE	not detected	113	> 100↑↑
Immunoglobulin heavy constant gamma 1	not detected	15	> 100↑
Immunoglobulin kappa constant	not detected	21	> 100↑
C3	not detected	28	> 100↑
Fibronectin	6	44	7.3↑

↑↑, marked increase; ↑, increase. Abbreviation: *Apo* apolipoprotein

Based on the abovementioned findings, the patient was diagnosed with LPG with a heterozygous ApoE-Sendai mutation (Arg145Pro).

**Fig. 3:**
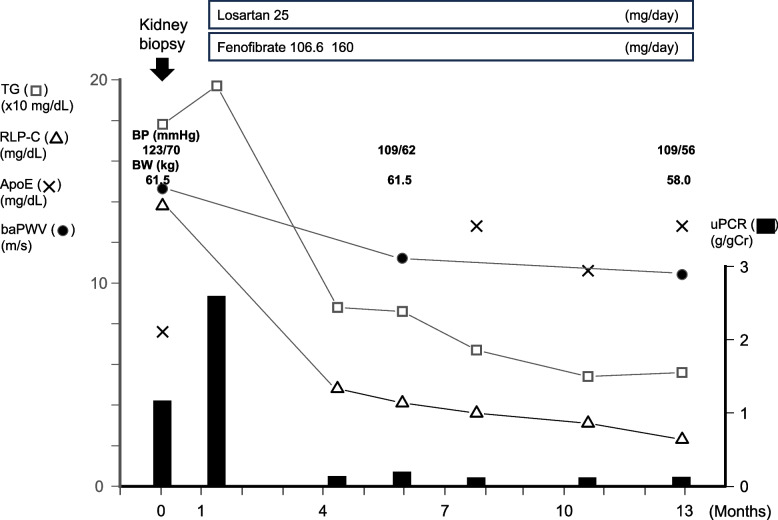
Clinical course of the patient. After the initiation of fenofibrate and losartan, improvements in TG and RLP-C levels along with a significant reduction in proteinuria were achieved. Moreover, a reduction in BP was achieved, and an improvement in baPWV was also attained. Furthermore, these effects were maintained despite a lack of improvement in the serum ApoE levels. Abbreviations: TG, triglyceride; RLP-C, remnant-like particle cholesterol; ApoE, apolipoprotein E; baPWV, brachial-ankle pulse wave velocity; uPCR, urine protein-to-creatinine ratio; BP, blood pressure; BW, body weight

We then commenced fenofibrate and losartan for the treatment of abnormal lipid profile and persistent proteinuria. Three months later, improvements in TG and remnant-like particle cholesterol (RLP-C) along with a significant reduction in proteinuria were achieved. Moreover, a reduction in BP, and an improvement in baPWV was also attained. Furthermore, these effects were maintained despite the lack of improvement in serum ApoE levels (Fig. 3, Table 1).

## Discussion and conclusion

Herein, we report the case of a patient with LPG with markedly increased arterial stiffness at the time of diagnosis, in whom combination therapy with fenofibrate and losartan successfully improved both proteinuria and arterial stiffness. To the best of our knowledge, this is the first case report of LPG in which the CVD risk was assessed using arterial stiffness.

LPG is histologically characterized by dilated glomerular capillaries with lamellated lipoprotein thrombi that lack foam cells [2, 3]. In the present case, the glomerular lesions, such as dilated capillary lumens with lipoprotein thrombi showed a laminated appearance without foamy macrophage infiltration and membranous nephropathy-like lesions. These findings were consistent with the known histologic features of LPG among ApoE-related glomerular diseases. Subsequent phenotype analysis by IEP, genotype analysis, DNA sequencing, and proteomics by LC–MS/MS revealed that the patient harbored a heterozygous ApoE-Sendai mutation.

No standard treatment regimen has been established for patients with LPG. However, LPG is typically associated with type III hyperlipidemia, and hypertriglyceridemia exacerbates LPG in humans and animal models [3]. Although different types of ApoE mutations have been reported [3], the efficacy of lipid-lowering agents, including fibrates, has been evaluated for each type of LPG mutation. Ieiri et al. reported a case of LPG with heterozygous ApoE Sendai mutation in which proteinuria was not detected 11 months after the initiation of a combined intensive lipid-lowering therapy comprising fenofibrate (300 mg/day), niceritrol (750 mg/day), ethyl-icosapentate (1800 mg/day), and probucol (500 mg/day) [14]. Arai et al. reported a marked improvement in nephrotic syndrome and disappearance of intraglomerular lipoprotein thrombi after two years of treatment with bezafibrate (400 mg/day) after switching from one year of pravastatin in a patient with LPG with a heterozygous ApoE2 Kyoto mutation [15]. Furthermore, Kinomura et al. reported the case of a patient with LPG with a heterozygous ApoE Okayama mutation whose proteinuria remarkably reduced within six weeks of commencing combined intensive lipid-lowering therapy with bezafibrate (400 mg/day) and ethylicosapentate (1800 mg/day) after substituting one month of pravastatin therapy [16]. As mentioned above, various therapeutic regimens, including fibrates, improve both clinical manifestations and histopathological lesions [14, 15]; therefore, fibrates are considered to play a central role in the treatment of LPG. Thus, treatment with fenofibrate and losartan was initiated in our case to improve the lipid abnormalities and reduce proteinuria. Shortly after initiation (three months later), improvements in TG and RLP-C levels along with a significant reduction in proteinuria were achieved. Moreover, these effects were maintained despite the lack of a decrease in serum ApoE levels (Fig. 3). The mechanisms responsible for the marked reduction in proteinuria in patients with LPG are currently unknown; however, the reduction in blood very-low-density lipoprotein levels by acting on the peroxisomal proliferator-activated receptor and activating lipoprotein lipase by fenofibrate [5], resulting in the reduction in renal lipid accumulation can be contemplated to be the primary mechanism. Furthermore, once the lipoprotein thrombi are reduced with fenofibrate, the antiproteinuric effect of losartan can be more effective.

Recently, the clinical course of his younger brother, an identical twin in this case, was reported [17]. The same ApoE mutation was observed in both cases. However, in contrast to the younger brother, the proteinuria was milder, and no vacuolated areas in the peritubular capillaries of the tubulointerstitium were observed in our case. Differences in serum ApoE levels at the time of kidney biopsy and/or in the non-genetic pathogenic mechanisms involved may underlie the differences in clinical and histopathological findings between the two cases.

Nevertheless, at the time of kidney biopsy, a marked increase in baPWV for his age was observed (14.3 m/s, equivalent to that of a 56-year-old man) (Fig. 3). However, to the best of our knowledge, there are no reports on the relationship between LPG and CVD risk, as assessed by arterial stiffness. Therefore, it may be difficult to discuss this issue from the perspective of ApoE mutation alone.

Arterial stiffness has been recognized as an indicator of arteriosclerosis and a predictor of cardiovascular events [9, 10]. Factors that increase arterial stiffness include age, BP, heart rate, diabetes mellitus, lipid metabolism, smoking, uric acid (UA) level, obesity, inflammation, and oxidative stress [9, 10]. In contrast to CAVI, baPWV is influenced by the BP at the time of measurement; however, our patient’s BP was normal at initial baPWV assessment, even without antihypertensive medication. Therefore, in our case, the factors responsible for markedly increased arterial stiffness may have been factors other than BP. Furthermore, the patient had never smoked, and his blood glucose, UA levels and BMI were within normal ranges. Therefore, the following mechanisms are considered to potentially underlie the markedly increased arterial stiffness observed at the time of kidney biopsy.

First, among the risk factors for baPWV [9], dyslipidemia may have contributed to the markedly increased baPWV at the time of kidney biopsy. In the context of lipid profile and its effects on baPWV, some studies have reported an association between serum TG levels rather than serum cholesterol levels and baPWV [9]. In addition, serum TG levels were an independent predictor of endothelial function [18], and Ryan et al. reported that fenofibrate reduced inflammation, improved markers of endothelial function and reduced arterial stiffness [19]. In our case, in addition to LDL, TG and RLP-C levels were higher at the time of kidney biopsy. Although the patient had no apparent history of dyslipidemia and his TG and RLP-C levels were not extremely high, after initiation of fenofibrate, baPWV improved with a decrease in TG and RLP-C, suggesting close involvement of hyperlipidemia in his markedly increased baPWV for his age at the time of kidney biopsy.

Second, the presence of the ApoE ε4 allele, which our patient carried, may be another factor contributing to his dyslipidemia and markedly increased arterial stiffness. Numerous studies have reported the relationship between ApoE genotype and CVD. The ApoE ε4 allele has specifically been associated with CVD risk [20]. The mechanisms underlying the associations between ApoE genotypes and CVD events are considered to include the variations in serum lipid concentration and inflammatory responses to different ApoE alleles [21]. As for ApoE genotype and plasma levels of major lipids, both ε2 and ε4 alleles are associated with unfavorable lipid profiles [22]. In the context of inflammation-related mechanisms, ApoE ε4 carriers reportedly have significantly lower and ApoE ε2 carriers have significantly higher levels of C-reactive protein than ApoE ε3/ε3 carriers [23]. Contrastingly, Gungor et al. reported significantly higher levels of lipoprotein-associated phospholipase A2 index, a vascular inflammation marker, in Apo E4 isoform carriers [24]. In addition, Tziakas et al. reported that ApoE ε4 carriers had lower levels of atheroprotective IL-10 [25]. **T**hese findings may support the possibility in patient carrying the ApoE ε4 allele that the net effect of the underlying disease status may contribute to future CVD via advanced arterial stiffness. The present case suggests that the CVD risk in patients with LPG can be reduced by therapeutic intervention, including the use of fenofibrate.

In conclusion, we describe the first case of LPG with markedly increased arterial stiffness at the time of diagnosis, in whom combination therapy with fenofibrate and losartan successfully improved both proteinuria and arterial stiffness. However, the significance of arterial stiffness and response to treatment in patients with LPG may vary according to the ApoE variant, and further case studies are needed.

## Data Availability

No datasets were generated or analysed during the current study.

## Abbreviations

LPG: Lipoprotein glomerulopathy
ApoE: Apolipoprotein E
CKD: Chronic kidney disease
CVD: Cardiovascular disease
baPWV: Brachial-ankle pulse wave velocity
LDL: Low-density lipoprotein
CAVI: Cardio-ankle vascular index
uPCR: Urine protein to creatinine ratio
RBC: Red blood cell
HPF: High-power field
eGFR: Estimated glomerular filtration rate
BP: Blood pressure
BMI: Body mass index
TG: Triglyceride
HDL-C: High-density lipoprotein cholesterol
DNA: Deoxyribonucleic acid
LC–MS/MS: Liquid chromatography-tandem mass spectrometry
RLP-C: Remnant-like particle cholesterol
UA: Uric acid
Apo: Apolipoprotein
Lp(a): Lipoprotein (a)
PAS: Periodic acid-Schiff
PCR: Polymerase chain reaction
